# Indispensable or toxic? The phosphate versus arsenate debate

**DOI:** 10.1111/1751-7915.12024

**Published:** 2012-12-27

**Authors:** M José Huertas, Carmen Michán

**Affiliations:** 1 Instituto de Bioquímica Vegetal y Fotosíntesis, Centro de Investigaciones Isla de la Cartuja, Universidad de Sevilla-CSICAv. Américo Vespucio 49, 41092, Seville, Spain; 2 Universidad de Córdoba, Campus de Rabanales, Dept. of Biochemistry and Molecular Biology, Edificio Severo Ochoa C-6, 2^a^Planta14071, Córdoba, Spain

Arsenic (As) is toxic, carcinogenic and causes serious health problems. While As occurs naturally due to volcanic activity, the major anthropogenic sources of As are metal processing, burning of coal and arsenic-based pesticides or herbicides. Arsenate (AsO_4_^3−^) and arsenite (AsO_3_^3−^) are the primary chemical forms found in soil. Because of the wide distribution of arsenic compounds, arsenic resistance is widespread among living organisms (Nordstrom, 2002). Most resistance systems reduce arsenate to arsenite and sequester it in a vacuole or expel it from the cell (Stolz *et al*., 2006). Intensive research has shed light on dissimilatory As (V) reduction and As (III) oxidation pathways.

Phosphate is essential to life. Living beings need phosphorus to function, along with other elements such as hydrogen, oxygen, carbon, nitrogen and sulfur. The phosphate ion, PO_4_^3−^, plays several essential cellular roles, such as maintaining the structure of DNA and RNA, combining with lipids to build cell membranes and transporting energy within the cell through the molecule adenosine triphosphate (ATP).

During the last two years, the metabolic antagonism that exists between arsenic and phosphorus in microbial metabolism has been the focus of intense study. Wolfe-Simon and colleagues (2011) revolutionized this area of research by describing the isolation of a bacterial strain, GFAJ-1, capable of thriving in a growth medium containing arsenate and lacking phosphate, hypothesizing that arsenic may substitute phosphorous to allow growth. GFAJ-1 was isolated from highly contaminated sediments and was able to grow in arsenate solution (at 60% the rate of growth in phosphate supplemented medium). Without arsenate or phosphate the strain was unable grow.

Thereafter, a number of published works have reported contradictory results to those of Wolfe-Simon *et al*. Last July, *Science* published two independent reports on the subject. The work performed by Professor Vorholt's group (Erb *et al*., 2012) used highly pure reactives combined elegantly with state-of-the-art chemical tracing techniques to monitor phosphate substitution by arsenate. The findings showed that phosphate is absolutely necessary for the growth of GFAJ-1, suggesting that previous results were misleading due to the presence of impurities in the chemicals used. Although arsenate was found to be combined to biomolecules as sugars or acids in the cultures, they demonstrate that these products are generated abiotically. Furthermore, the authors found no evidence of arsenate incorporation into DNA or other core metabolic molecules. Similar conclusions were drawn by Dr Redfield's group (Reaves *et al*., 2012). Their findings showed that the GFAJ-1 strain grows in the complete absence of arsenate and that phosphate is absolutely necessary. Again, chemical impurities are named as the cause of conflicting results of the original GFAJ-1 report (Wolfe-Simon *et al*., 2011). Dr Redfield's group also investigated the putative presence of arsenate in DNA, while doing so using an alternative experimental method. The authors postulated that arsenate incorporation in DNA would result in higher instability. They electrophoretically tested hydrolysis of chromosomal DNA isolated from GFAJ-1 cultures grown under phosphate or arsenate and were unable to detect any significant amount of As in the nucleic acids. Consequently arsenate does not appear to act as a replacement for phosphate in DNA within this strain.

Taken together, these results, while not as exceptional as originally thought, still position GFAJ-1 as a strain of great value by virtue of its ability to tolerate arsenate. Thus, the mechanism through which this is achieved represents a highly worthwhile area of study.

Of most importance to this topic is the question of how the GFAJ-1 strain is able to glean enough phosphate in the arsenate-rich environment from which it was originally isolated. A mechanistic explanation had been published in *Nature* (Elias *et al*., 2012) where the authors studied a set of phosphate-binding proteins (PBP) that are part of the transport chain bacteria use to uptake phosphate from the environment. The ability of different PBPs to discriminate between phosphate and arsenate was investigated, finding that most had a 500- to 850-fold higher selectivity for phosphate. Even more impressive, GFAJ-1's PBP-2, which is upregulated in low-phosphate conditions, showed a 4500-fold selectivity for phosphate over arsenate. The observed differences in phosphate selectivity are related to the binding of anions to these proteins, which form a dense and rigid network of ion-dipole interactions. This network is sensitive to geometric changes. The authors found, through X-ray crystallography, that there are differences in alignment of one of the hydrogen bond angles in the binding pocket when PBPs bind arsenate, allowing them to select phosphate over arsenate even in highly arsenate-rich environments.

Additionally, Basturea and colleagues (2012) provide a simple explanation for how phosphorous may be obtained in arsenate-rich or phosphate-limiting environments. They show that, under these conditions, ribosome degradation is induced, which releases free bases from RNA and provides sufficient phosphate to allow limited growth.

Together with arsenate versus phosphate discrimination and the ability to obtain phosphorus, it would be expected that a resistance mechanism preventing the intracellular accumulation of As would be important for the survival of the GFAJ-1 strain in high-arsenate and phosphate-limiting conditions. In studies that relate to this, Lopez-Maury and colleagues (2009) constructed a *Synechocystis* mutant in which all genes encoding arsenate reductases were inactivated. The strain was more sensitive to arsenate in an arsenate-rich medium devoid of phosphate than in a medium with phosphate (where the mutant grows similarly to wild type), suggesting that small amounts of As (V) enter the cells under P-limiting conditions.

A twist in the phosphate versus arsenate debate was brought to the forefront in recent *Environmental Microbiology* article. In *Agrobacterium tumefaciens,* a model microorganism used to study the genes involved in As (III) oxidation, genes related to arsenate oxidation (*aioBA* and *aioSR*) are adjacent to genes involved in phosphorous acquisition (*pst/pho*) (Kang *et al*., 2012), suggesting that they may be functionally related. Moreover, *aio* genes are expressed in response to phosphate starvation. In line with this, the manuscript shows that key genes related to As (III) oxidation and Pi stress are co-regulated by ArsR1, an ArsR-type repressor that controls the arsenic detoxification system. Furthermore, a link between the presence of environmental phosphate on the metabolism of other nutrients, including arsenic, is revealed.

A key limitation of the above studies is the uncertain purity of the chemicals used. This uncertainty leads to the possibility that cross-contamination between arsenite and phosphate may have occurred. As arsenic and phosphorous are very close in the periodic table, separating them chemically is not easy. On the other hand, in complex natural environments, chemical methods cannot measure other limiting factors such as bioavailability, and therefore future studies should aim to use biologically based measurement methods for this purpose. One possible biological system for arsenite detection was reported in *Microbial Biotechnology* (Wackwitz *et al*., 2006). The manuscript describes the construction of different recombinant strains using the ArsR sensor from *Escherichia coli* to control transcription from two very different reporter genes: the well-known *lacZ* coding for β-galactosidase and the *Saccharomyces cerevisiae* cytochrome peroxidase *ccp* gene. Combination of both reporters provides an extremely easy and fast method for semi-quantitative detection of As with a threshold as low as 4 μg l^−1^. This system represents an excellent model for the development of biosensors capable of to detecting rare elements both in laboratory solutions and in the environment.

A final related topic that deserves more detailed study are the microbial processes that, together with inorganic and physical mechanisms, contribute to the global arsenic cycle. The environmental factors that drive arsenic mobilization and the influence of microbial As metabolism in the environment should be a key focus of future studies.

## Conflict of interest

None declared.

## Funding Information

No funding information provided.
